# Cathepsin D Expression in Colorectal Cancer: From Proteomic Discovery through Validation Using Western Blotting, Immunohistochemistry, and Tissue Microarrays

**DOI:** 10.1155/2012/245819

**Published:** 2012-08-07

**Authors:** Chandra Kirana, Hongjun Shi, Emma Laing, Kylie Hood, Rose Miller, Peter Bethwaite, John Keating, T. William Jordan, Mark Hayes, Richard Stubbs

**Affiliations:** ^1^Wakefield Biomedical Research Unit, University of Otago, Wellington 6242, New Zealand; ^2^School of Medicine and Health Sciences, University of Otago, Wellington 6242, New Zealand; ^3^Department of Pathology and Molecular Medicine, University of Otago, Wellington 6242, New Zealand; ^4^Capital & Coast District Health Board, Wellington Hospital, Wellington 6021, New Zealand; ^5^Centre for Biodiscovery, School of Biological Sciences, Victoria University of Wellington, Wellington 6012, New Zealand

## Abstract

Despite recent advances in surgical techniques and therapeutic treatments, survival from colorectal cancer (CRC) remains disappointing with some 40–50% of newly diagnosed patients ultimately dying of metastatic disease. Current staging by light microscopy alone is not sufficiently predictive of prognosis and would benefit from additional support from biomarkers in order to stratify patients appropriately for adjuvant therapy. We have identified that cathepsin D expression was significantly greater in cells from invasive front (IF) area and liver metastasis (LM) than those from main tumour body (MTB). Cathepsin D expression was subsequently examined by immunohistochemistry in tissue microarrays from 119 patients with CRC. Strong expression in tumour cells at the IF did not correlate significantly with any clinico-pathological parameters examined or patient survival. However, cathepsin D expression in cells from the MTB was highly elevated in late stage CRC and showed significant correlation with subsequent distant metastasis and shorter cancer-specific survival. We also found that macrophages surrounding tumour cells stained strongly for cathepsin D but there was no significant correlation found between cathepsin D in macrophages at IF and MTB of CRC patient with the clinic-pathological parameters examined.

## 1. Introduction

Despite advances in surgical techniques and therapeutic interventions during the past few decades, colorectal cancer (CRC) remains a major health problem worldwide. The American Cancer Society estimated that some 141,210 people would be diagnosed with colorectal cancer in the US in 2011 and that one-third of them would die of the disease [1]. In New Zealand around 2800 individuals are diagnosed with CRC annually and nearly half of them will die as a result of the disease [2]. Most deaths will result from metastatic spread, most commonly to the liver. Death from CRC is preventable by surgery alone in its early stages [3]. Adjuvant chemotherapy, which aims to eradicate subclinical tumor deposits after surgical removal of the primary tumor, has been shown to reduce tumor recurrence and improve disease-free survival. While the use of adjuvant chemotherapy for stage III CRC patients has become standard practice, its application for stage II patients is more controversial [4].

Current histological staging methods by light microscopy alone are not sufficiently accurate to predict metastatic spread as there is significant variation with respect to clinical outcomes within currently used stages. Thus, some 20–30% of stage II patients will develop metastases and die of their disease, and some 30% of stage III patients will not develop recurrent disease even without adjuvant chemotherapy [4]. Discovery of additional prognostic markers might permit the development of guidelines for better management of CRC in order to improve overall survival. Modern proteomics provides us with the tools to discover new, potentially valuable biomarkers.

Cathepsin D is an aspartic lysosomal endopeptidase present in most mammalian cells. Overexpression of this protease has been associated with the progression of several human cancers including gastric carcinoma [5–7], melanoma [8], and ovarian cancer [9]. Cathepsin D has been comprehensively studied in breast cancer where overexpression of mRNA and protein has been observed [10, 11] and been shown to be an independent marker of poor prognosis [12, 13]. Cathepsin D levels in tumors were reported to be higher than in adjacent normal tissue [14, 15]. The role of cathepsin D in cancer has been postulated to promote tumor growth directly by acting to degrade and remodel the basement membrane and interstitial stroma surrounding the primary tumor [16] and indirectly by stimulation of other enzymes or in cooperation with other cathepsins in the proteolysis process [17]. Previous reports on the clinical significance of cathepsin D in CRC have been variable and inconsistent. On the one hand, cathepsin D expression in tumor and stromal cells at the IF region has been reported to significantly correlate with lymph node metastasis [18] and hence survival. However, another group has reported a study in 48 patients with CRC in which expression of cathepsin D did not differ between MTB and the IF [19].

We used laser microdissection to isolate proteins from CRC tumor cells taken from main tumor body (MTB), invasive front area (IF), and liver metastasis (LM) and then profiled and compared proteins using saturation label dye 2D-DIGE. The concentration of cathepsin D was found to be elevated in tumor cells at the IF area and LM compared to cells at the MTB in tissue from the same patients. This paper explores the expression of cathepsin D in CRC tissue using immunohistochemistry to explore its potential value as a biomarker of metastasis.

## 2. Material and Methods

### 2.1. Identification of Overexpression of Cathepsin D

#### 2.1.1. Tissue Samples

Primary colorectal tumor and LM from the same patient were collected from eight patients with sporadic CRC undergoing surgery at Wakefield Hospital, Wellington, New Zealand, and used for proteomic analysis. (See Table 1 for patients' clinico-pathological features.) Tumor specimens were collected directly from the operating theatre and immediately snap-frozen in liquid nitrogen and stored at −80°C. Written, informed consent was obtained from all patients participating in this study and ethics approval was given by the Central Regional Ethics Committee, in accordance with the Helsinki Declaration of 1975.

#### 2.1.2. Discovery Phase

We have used a combination of laser microdissection, saturation labeling 2D DIGE, and MALDI TOF mass spectrometry to compare the protein expression profiles of the main tumor body (MTB), invasive front (IF), and liver metastasis (LM) in sets of tissues from 8 CRC patients, in a biomarker discovery program. The methods in this process have previously been described [20, 21] except that saturation labeling rather than minimal labeling was used. Through this process we identified that cathepsin D was upregulated at both the IF and LM compared to the MTB suggesting that it may play a role in metastasis.

### 2.2. Validation of Overexpression of Cathepsin D in the Invasive Front and Metastases

#### 2.2.1. Western Blotting

Saturation-labeled proteins were separated by 2DE as described previously [21] and transferred to PVDF transfer membrane (Amersham Hybond LFP, GE Healthcare, Sweden) at 50 V for 4 h (Bio-Rad, USA) in transfer buffer (3% (w/v) Tris, 14.4% (w/v) glycine, 20% methanol). The blot was blocked overnight in 5% ECL blocking solution (GE Healthcare), rinsed in Tris-buffered saline (TBS) (137 mM NaCl, 20 mM Tris, pH 7.2) containing 0.1% (v/v) Tween-20 (T-TBS), and incubated with a mouse monoclonal antibody [CTD-19] to cathepsin D (Abcam, Cambridge, UK) in T-TBS for 2 h at RT. The blot was rinsed 3 times for 10 min in T-TBS and incubated with an ECL-Plex Cy3-conjugated goat anti-mouse antibody (GE-Healthcare) at 1 : 2500 diluted in T-TBS. The blot was rinsed three times for 10 min in T-TBS, once in TBS, and dried in the dark overnight. Blots were scanned with the Cy3 and Cy5 channel of the Fujifilm FLA-5100 and images overlaid using ImageQuant (GE Healthcare). The primary antibody, mouse anti-cathepsin D antibody condjugated to HRP (CTD-19, ab6313, Abcam, Cambridge, UK) at 1 : 200 dilution was used directly on the gel and detected using ECL-Plus (GE Healthcare) on the Cy2 channel of the Fujifilm FLA5100.

#### 2.2.2. Immunohistochemistry

Immunohistochemistry (IHC) was undertaken on formalin-fixed paraffin-embedded (FFPE) tumor specimens. Stains were carried out for cathepsin D, CD45 as a leukocyte marker, and CD68 as a macrophage marker. Sections were deparaffinized in xylene and rehydrated in decreasing concentration of ethanol (100%, 90%, 80%, 70%). Antigen was retrieved using 10 mM sodium citrate buffer (pH 6.8) for 10 min in pressure cooker. Slides were incubated in 3% H_2_O_2_, 50% methanol in wash buffer (phosphate-buffered saline (PBS) (Oxoid), 0.1% Tween-20) for 10 min to quench endogenous peroxidases. Primary antibodies were incubated for 1 h at RT. The ImmPRESS Universal kit (Vector Laboratories, CA, USA) was used to detect primary antibodies and developed with DAB or Nova Red substrate (Labvision, CA, USA). Sections were counterstained with haematoxylin, dehydrated, and mounted. Primary antibodies used in IHC, which were purchased from Abcam (Cambridge, UK), were mouse monoclonal anti-cathepsin D conjugated with HRP (CTD-19, ab6313) at 1 : 7000 dilution, mouse monoclonal anti-CD45 (MEM-28, ab 8216) at 1 : 1000 dilution, and mouse monoclonal anti-CD68 (KP1, ab 955) at 1 : 400 dilution.

#### 2.2.3. Patient Sample for Tissue Microarray

Out of the 282 patients who had undergone partial colectomy or anterior resection of CRC performed by Dr. John Keating from 1997 to 2005 at Wellington Hospital, 169 patients had archival tissue blocks available for investigation at the time of this study. A representative block from each patient was drawn and sectioned for H&E staining. On histological examination, 42 blocks were excluded from the cohort due to the absence or inadequacy of tumor cells in the sections from the blocks initially chosen from the tissue archives. Consequently a total of 127 CRC cases were finally included in this study. Of the patients chosen, 26 had received chemotherapy (17 × 5FU + leucovorin, 1 oxaliplatin, 1 capecitabine, 2 5FU + mitomycin C, 4 5FU infusion), 13 had received radiotherapy and 6 had received chemoradiation. As chemotherapy and radiation therapy are common treatments before surgery all treated patients were included in the test cohort.

Clinicopathological features of the resected CRC were obtained from a prospective database maintained by Dr. John Keating according to the clinical and pathological reports held at Wellington Hospital. Pathological stages were classified according to the TNM staging system. Histological grading and typing of the tumor were determined according to the World Health Organisation tumor classification system. Cancer-specific survival was defined as the interval between the date of the first operation of the primary tumor to the date when the patient died from recurrent CRC. Cases were censored at the end of the followup or at the time of death due to other causes. Thirty-seven patients died of recurrent CRC during the follow-up period. Medium follow-up time was 61 months (ranging from 2 to 164 months). Construction of tissue microarrays (TMAs) and immunohistochemistry on the TMAs using archival human tissues was conducted with the approval of the New Zealand Central Regional Ethics Committee.

#### 2.2.4. Tissue Microarray Immunohistochemistry

Five tissue microarray blocks (TMA) containing a total of 127 CRC cases were constructed. Each TMA consisted of up to 26 tissue cores with a single tissue core per patient's tumor. Formalin-fixed, paraffin-embedded tissue blocks of CRC were obtained from the hospital tumor archive. Before constructing TMAs, a 4 *μ*m section was sliced from each tumor block for a routine H&E inspection by a pathologist. After histological confirmation of the tumors, areas of sampling (AOS) were defined and marked on the microscope slide by the pathologist. These microscope slides with spotted AOS were used later to guide the location of tissue cores for punching. AOS was defined as the area of obvious invasive cancer closest to the lumen, not including any potential adenomatous areas. TMAs were constructed using the Beecher automated tissue arrayer (ATA-27, Beecher Instruments, Sun Prairie, Wisconsin, USA) through the Molecular and Clinical Pathology Research Laboratory, Clinical and State-wide Services, Princess Alexandra Hospital, Queensland, Australia. A tissue core with a diameter of 1 mm was punched from the donor tissue under the guidance of AOS, and transferred to a recipient paraffin block (array margin of 10 × 20 mm). Once the TMAs were made, they were heat cycled from 60°C for 1 h and room temperature (RT) for 1 hr for a total of 5 cycles to aid cutting.

#### 2.2.5. Scoring of IHC in TMAs

Expression of cathepsin D and CD68 was graded and scored by two blinded independent pathologists (PB and RM). The intensity of cathepsin D at the MTB and IF was scored from 1 to 3, with 1 for weak or none, 2 for moderate, and 3 for strong. The presence of macrophages, stained with CD68, at the MTB or IF was scored from 1 to 3, with 1 for scanty or none, 2 for moderate, and 3 for plentiful. These were scored separately in order to identify whether expression of cathepsin D was in the tumor cells of the invasive front or in macrophages associated with the invasive front. This expression was then correlated with clinical parameters.

#### 2.2.6. Statistical Analysis

Statistical analyses were performed using SPSS (version 17). The association between cathepsin D and CD68 immunoreactivity scores and patient clinico-pathological parameters was assessed by *χ*-square test. The impact of cathepsin D and CD68 on patient survival was examined by Kaplan-Meier analysis and the statistical significance determined by log-rank test. A multivariate analysis based on Cox proportional hazard regression model was applied to determine independent prognostic factors. Variables included in Cox regression analysis were histological grade, histological types, Dukes stage, vascular invasion, perineural invasion, type of operation, distant metastasis, and cathepsin D and CD68 immunoreactivity scores. A *P* value less than 0.05 was considered statistically significant.

## 3. Results

**Figure 1:**
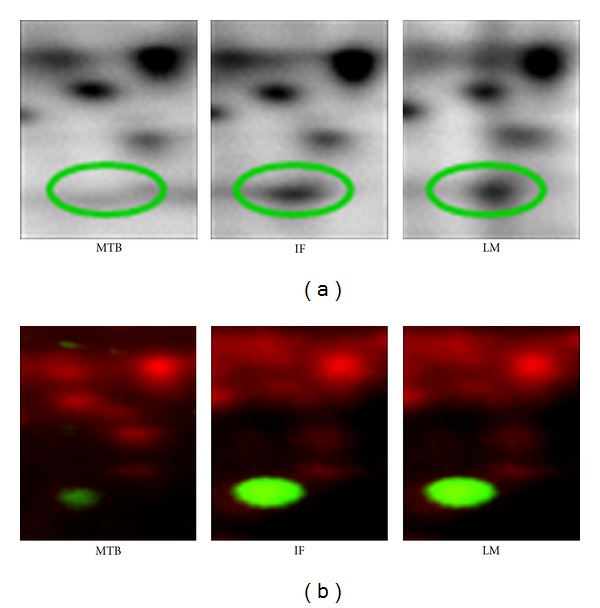
(a) Spots of cathepsin D from samples of the main tumor body (MTB), IF area (IF), and liver metastasis (LM) on 2 D gels; (b) validation of cathepsin D profiled by 2DE western blotting of cy5 labeled LMD sample (red) and using Cy3 label antibody (green) (ECL-Plex).

**Figure 2:**
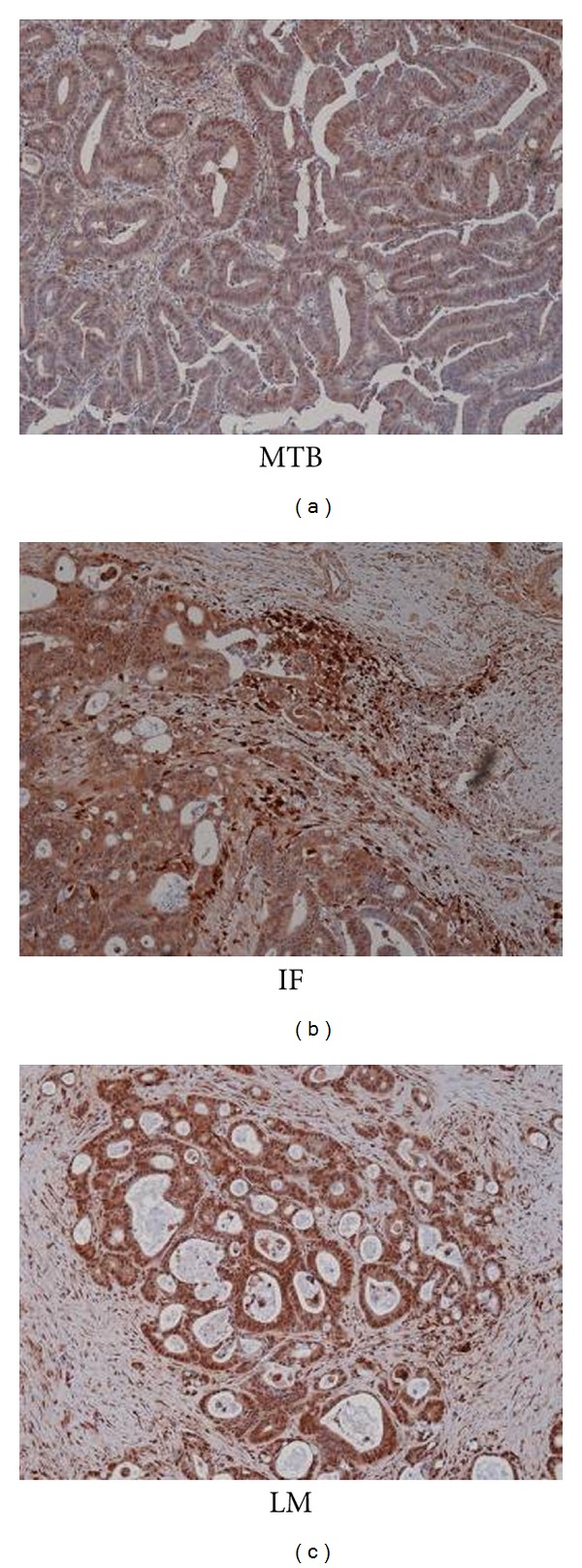
Validation of cathepsin D expression at two different regions, main tumor body. (MTB) and invasive front (IF) area of primary tumor and liver metastasis (LM) from the same patient by immunohistochemistry (IHC) (DAB substrate, brown). Sections were counterstained with haematoxylin (blue) (20x objective).

Cathepsin D was found to have different expressions in three different areas of interest of colorectal tumor tissues in our discovery project. Expression of cathepsin D in tumor cells at the IF and in LM was significantly higher than that in the MTB when profiled using 2 D-DIGE (Figure 1(a)). The gel spot position and concentration of cathepsin D in these three areas of tumor were validated with 2DE western blotting (Figure 1(b)). Validation by IHC also confirmed relatively greater abundance of cathepsin D at the IF and in LM compared to MTB in 9 of 11 (82%) CRC patients. IHC images from a representative set of tissues from the same patient are presented in Figure 2.

**Figure 3:**
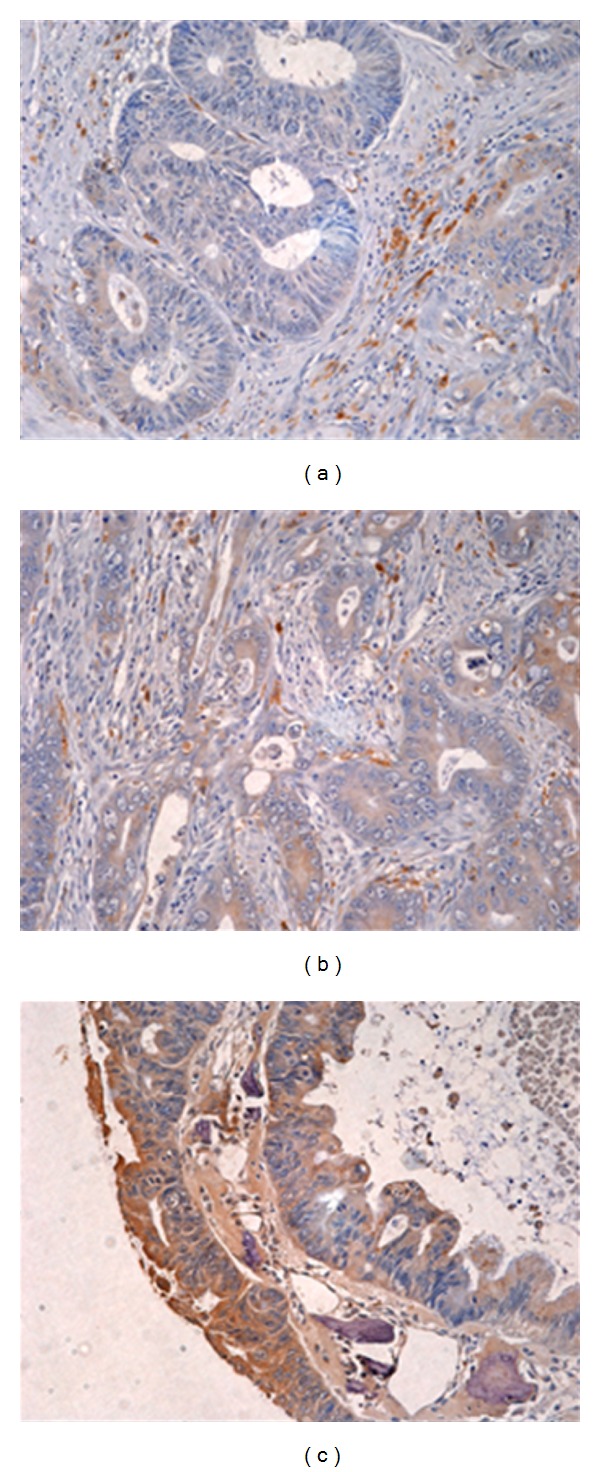
Representative CRC tissue for intensity scoring of tissue microarray immunohistochemistry (DAB substrate, brown). None or weak expression of cathepsin D (a) was scored 1. (b) Moderate expression of cathepsin D in tumor cells was scored 2 and strong expression of cathepsin D (c) was scored 3. Tissue sections were counterstained with haematoxylin (blue) (20x objective).

Following the creation of TMAs from 127 patients with CRC, tissue damage or loss occurred in 8 patients which left 119 patients available for our validation study. Examples of cathepsin D expression in tissues for TMA IHC for scoring purposes are presented in Figure 3.

High-level expression of cathepsin D at the IF area of colorectal tumor did not correlate significantly with any of the clinico-pathological parameters examined (data not shown). Cathepsin D expression in tumor cells of the MTB did however correlate significantly with distant metastases (*P* = 0.038) and tended to correlate with TNM stage although this did not reach statistical significance (*P* = 0.064). Cathepsin D expression in the MTB did not correlate with age, gender, tumor location, histological type and grade, vascular invasion, perineural invasion, nodal status, or depth of invasion. This data is shown in Table 2.

**Figure 4:**
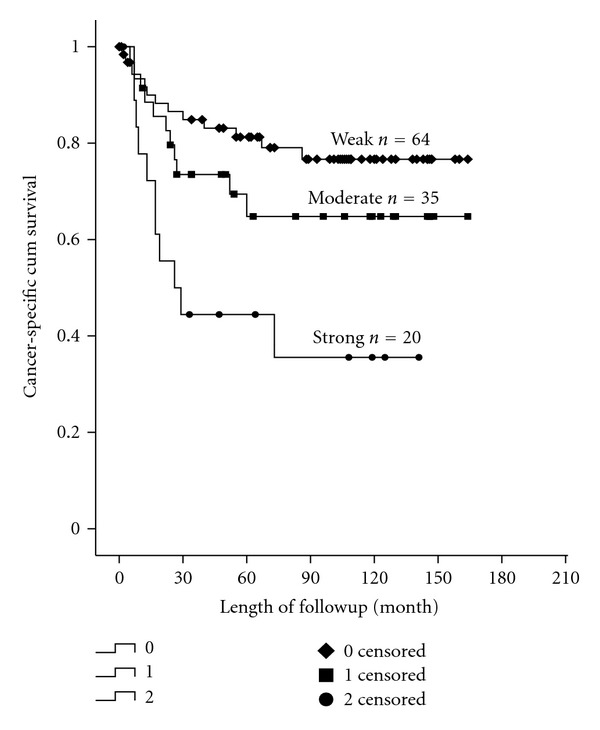
Cancer-specific survival (in months) of CRC patients in association with cathepsin D expression in epithelial of main tumor.

Expression of cathepsin D in the MTB was inversely correlated with 5-year cancer-specific survival in univariate analysis using Kaplan-Meier statistics and log-rank test. Those with strong expression of cathepsin D had a 5-year cancer-specific survival of 42%, compared with 63% for those with moderate expression (*P* = 0.039) and 81% for those with weak expression (*P* = 0.0001). 5-year cancer-specific survival was not significantly different between those with weak expression of cathepsin D and those with moderate expression (*P* = 0.198) (Figure 4).

Multivariate Cox regression analysis revealed that cathepsin D is not an independent prognostic factor (*P* = 0.958) unlike TNM stage (*P* = 0.0001) and perineural invasion (*P* = 0.039) (Table 3), which are known to be independent prognostic factors in CRC.

**Figure 5:**
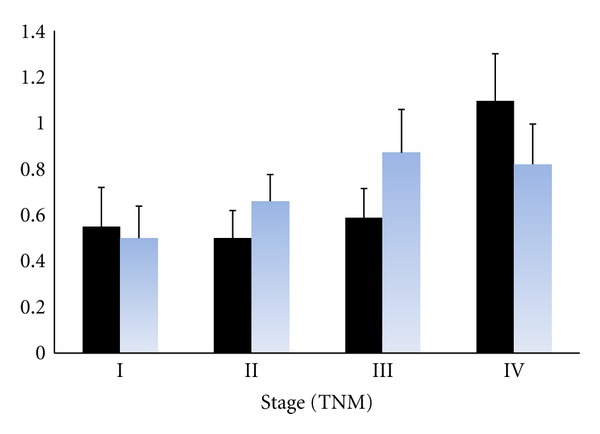
The average score of cathepsin D expression in tumor cells at the MTB (solid black) and IF (blue) of CRC patients at different stage (TNM).

The average immunoreactivity score (score ± SE) of cathepsin D at different stages of CRC is shown in Figure 5. Cathepsin D scores were similar for stage I, II, and III patients but were significantly higher in the patients with stage IV disease. The score of cathepsin D is approximately double in stage IV patients compared to those with earlier stage disease.

**Figure 6:**
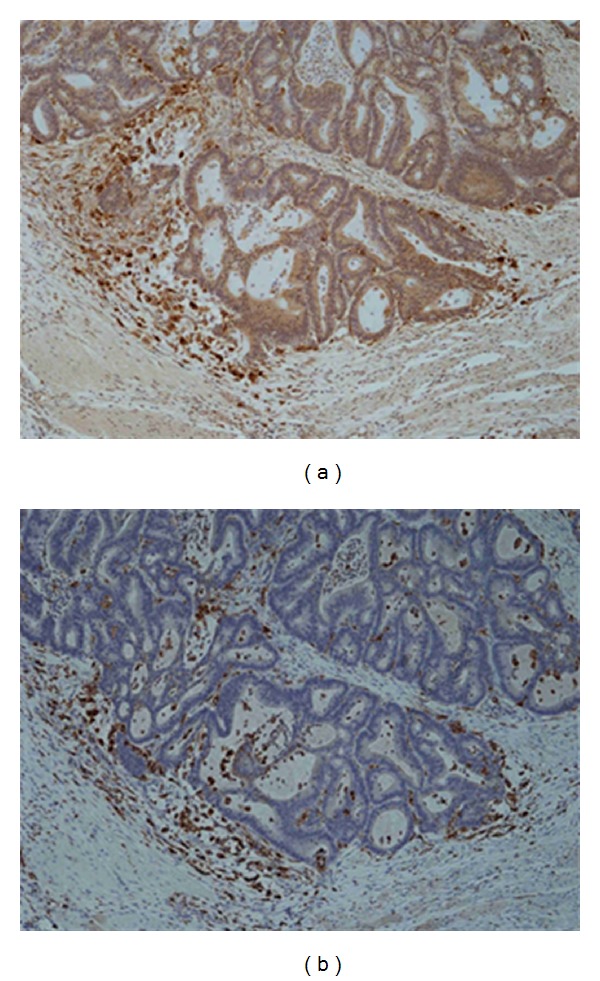
Adjacent sections of primary colorectal tumor stained for cathepsin D (a) (DAB substrate, brown). CD 68 staining (b) confirmed that very strong expression of cathepsin D in a population of cells within stromal tissue was associated with macrophage (DAB substrate, brown). Sections were counterstained with haematoxylin (blue) (40x objective).

Strong cathepsin D staining was noted in a population of cells within stromal tissue at the IF during validation with IHC. Immunohistochemistry of adjacent sections for CD45, a leukocyte common antigen, confirmed that cells staining for high levels of cathepsin D also stained for CD45, confirming that they were leukocyte phenotype rather than cancer cells. Using CD68 (a specific marker of monocytes/macrophages), it was demonstrated that the cells at the IF most strongly staining for cathepsin D were of monocytes/macrophage phenotype rather than being cancer cells (Figure 6). The presence of macrophages containing cathepsin D at the IF area of colorectal tumor did not correlate significantly with any of the clinico-pathological parameters examined (data not shown).

## 4. Discussion

The depth of invasion of colorectal cancer through the bowel wall and the presence or not of lymph node involvement have provided the basis for pathological staging since it was first described by Dukes some 75 years ago. However useful this is, we know the allocation of patients to stage II (Dukes B) or stage III (Dukes C) disease carries an imprecise estimate of prognosis. This has become problematic following the widespread adoption of adjuvant chemotherapy, as it is now important to identify as accurately as possible those patients who despite a complete surgical clearance of the primary tumor, will develop recurrent or distant disease. There is optimism that molecular markers may be discovered which would help refine assessments of prognosis, allow more precise allocation of adjuvant therapies, and perhaps even point to new drug targets for this disease. Modern proteomics has the capacity to identify such molecular biomarkers. In this paper we briefly describe the initial approach taken by our group, using laser microdissection to precisely identify CRC tissue and specific areas of CRC, for further proteomic analysis using 2D DIGE and MALDI-TOF mass spectrometry.

The IF area of colorectal cancer has been suggested as a critical interface where tumor progression and metastasis begin and may therefore be a critical area in which to search for prognostic markers. Areas of tumor budding have been shown to have overexpressed proteins which are involved in extracellular matrix degradation [22]. Proteins including matrix metalloproteinase-9 (MMP9), cathepsin B [23], matrilysin, and laminin [24] have been identified as being highly expressed in tumor budding. For this reason we were interested in examining and comparing the proteome derived from the MTB and the IF of the primary CRC, and the proteome derived from liver metastases all in the same patient. By so doing we identified that cathepsin D was expressed more strongly at the IF area in both tumor cells and what we identified to be macrophages surrounding tumor glands compared to its expression in the MTB.

Our examination of cathepsin D at the IF by immunohistochemistry suggested that much of the expression in that area was not associated with tumor cells themselves but with what appeared to be leucocytes or macrophages. Using a leukocyte common antigen marker (CD45) we established that much of the positive staining related to leukocytes at the IF, but that not all the leukocytes stained for cathepsin D. Using a specific monocyte/macrophage marker (CD68) we identified that the majority of the cathepsin D staining at the IF was in macrophages which may be considered a marker of the immune response to the tumor. Interestingly, Brujan et al. (2009) [25] also found macrophage-like cells surrounding breast cancer cells which contained strongly positive cathepsin D granules.

Nadji et al. (1996) [26] reported that cathepsin D in stromal cells significantly correlated with disease free and survival but not in tumor cells when examined in node-negative breast cancer patients. Similarly Theodoropoulos et al. (1997) [27] reported that positive cathepsin D staining in stromal cells and negative cathepsin D in tumor cells showed worse prognosis in 60 CRC patients. They suggested that positive CD expression in stromal cells may be used as an important indicator of tumor progression.

Having identified that the cathepsin D staining at the IF could be in either tumor cells or macrophages, we scored the cathepsin D expression at the IF in our TMAs separately in the tumor cells and in the macrophages. Neither cathepsin D expression in tumor tissue nor that in macrophages at the IF significantly correlated with any of the clinico-pathological parameters examined in our 119 patients. Guzińska-Ustymowicz et al.found no correlation between tumor budding and the activity of cathepsin D expression [28]. They concluded that cathepsin D in the tumor cells at the IF area was not involved in tumor progression and metastasis in CRC. On the other hand, the expression of cathepsin D at the MTB was found to be significantly associated with distant metastases and the correlation with TNM stage approached statistical significance (*P* = 0.064). We noted that expression of cathepsin D was highly elevated in late-stage CRC patients (TNM stage IV) compared to the earlier stages (TNM stages 1I, II, and III). Mayer et al. (1997) [29] noted findings similar to results of our study. They have found that cathepsin D was only elevated in CRC patients with Duke's C and D. They found that the elevation of cathepsin D was not significantly correlated with the clinicopathological parameters examined.

A role for cathepsin D in cancer metastasis was first demonstrated in an *in vitro* study using rat tumor cells in which overexpression of procathepsin D was associated with metastatic potential [30]. The concentration of cathepsin D in chronic ulcerative colitis and familial adenomatous polyposis, which is known to associate with the increase risk of colorectal carcinoma and colon carcinoma, was higher than that of normal colon [31]. Cathepsin D has been postulated to be secreted from cancer cells and been shown to serve as an autocrine growth factor in several cancer studies conferring proinvasive and prometastatic properties [32]. When our results are taken alongside those of others [18, 22, 28], one is forced to conclude that any role of cathepsin D in CRC progression remains uncertain.

The role of tumor infiltrating lymphocytes (TILs) especially macrophages in solid tumors remains unclear as they have been implicated in both tumor progression and protective host response. There are studies that have reported that pronounced tumor infiltration with TILs is associated with early-stage disease and/or improved survival [33], and yet other compelling evidence has emerged recently to indicate that tumor-associated macrophages (TAMs)—also referred to as alternative M2 macrophages—have an important role in solid tumor progression [34]. In our present IHC microarray study, neither strong expression of cathepsin D in macrophages nor the abundance of macrophages themselves at the IF of colorectal cancer tissue correlated particularly with other important clinico-pathological parameters, survival or metastasis.

In the discovery phase of our study cathepsin D was more highly expressed in LM and at the IF of CRC, relative to the MTB, suggesting it might be associated with tumor progression. When cathepsin D expression was examined in TMAs from 119 patients with CRC, the higher expression at the IF relative to the MTB was confirmed, but this was largely related to its presence in macrophages rather than tumor cells *per se*. However, neither expression of cathepsin D in the CRC cells at the IF nor the presence of cathepsin D staining macrophages at the IF correlated well with other clinico-pathological parameters examined. Paradoxically strong expression of cathepsin D in the cells of the main tumor body was noted in late-stage disease and significantly correlated with distant metastasis and shorter cancer-specific survival. Cathepsin D expression in the main tumor body warrants further consideration as a potential biomarker of prognosis in colorectal cancer.

## Figures and Tables

**Table 1:** Clinico-pathological details of patients.

	Patient 1	Patient 2	Patient 3	Patient 4	Patient 5	Patient 6	Patient 7	Patient 8
Site of primary tumor	Recto-sigmoid	Caecum	Ascending colon	Sigmoid colon	Recto-sigmoid	Caecum	Ascending colon	Recto-sigmoid
Degree of differentiation	Moderately	Poorly	Poorly	Moderately	Moderately	Poorly	Moderately	Well
Age at diagnosis^∗^	58	71	63	81	50	54	58	65
Gender	F	M	F	M	M	M	F	M
TNM stage of primary tumor	2	3	3	2	3	3	3	2
LM diagnosis^∗∗^	Met	Synch	Synch	Synch	Synch	Synch	Synch	Synch
Liver involvement^∗∗∗^	Solitary <25%	Multiple 25–50%	Multiple >50%	Multiple 25–50%	Three 25–50%	Multiple >50%	Multiple <25%	Four <25%

^∗^Mean age at primary tumor diagnosis: 62.9 ± 10 (mean ± SD).

^∗∗^Met: Metachronous diagnosis, Synch: Synchronous diagnosis.

^∗∗∗^Number of liver metastases (multiple: too many to count), plus percentage of liver involvement.

**Table 2:** Correlation of cathepsin D expression at MTB with clinico-pathological features.

Clinico-pathological parameters	No. of cases (%)^1^	neg/weak (%)^2^	Moderate (%)^2^	Strong (%)^2^	*P* value^3^
Age					0.139
<65	35 (29)	14 (40)	14 (40)	7 (20)	
≥65	84 (71)	50 (60)	21 (25)	13 (15)	
Gender					0.586
F	59 (50)	30 (51)	17 (29)	12 (20)	
M	60 (50)	34 (57)	18 (30)	8 (13)	
Tumor location					0.072
Colon	82 (69)	49 (60)	19 (23)	14 (17)	
Rectum	37 (31)	15 (41)	16 (43)	6 (16)	
Histological type					0.177
Nonmucinous	102 (86)	52 (51)	33 (32)	17 (17)	
Mucinous	16 (14)	12 (75)	2 (13)	2 (13)	
Histological grade					0.211
High grade	95 (81)	47 (50)	31 (33)	17 (18)	
Low grade	23 (19)	16 (70)	4 (17)	3 (13)	
Vascular invasion					0.400
Negative	90 (76)	51 (57)	26 (29)	13 (14)	
Positive	29 (24)	13 (45)	9 (31)	7 (24)	
Perineural invasion					0.839
Negative	109 (92)	59 (54)	32 (29)	18 (17)	
Positive	9 (8)	4 (44)	3 (33)	2 (22)	
TNM stages					**0.064**
I	20 (17)	12 (60)	5 (25)	3 (15)	
II	42 (36)	27 (64)	10 (24)	5 (12)	
III	34 (29)	18 (53)	13 (38)	3 (9)	
IV	21 (18)	7 (33)	6 (29)	8 (38)	
Distant metastasis					**0.038** ^ ∗^
No	89 (75)	52 (58)	27 (30)	10 (11)	
Yes	29 (25)	12 (41)	8 (28)	9 (31)	
Nodal status					0.358
Negative	66 (58)	38 (58)	16 (24)	12 (18)	
Positive	47 (42)	24 (51)	17 (36)	6 (13)	
Depth of invasion					0.935
T1/T2	23 (20)	13 (57)	7 (30)	3 (13)	
T3/T4	93 (80)	51 (55)	27 (29)	15 (16)	
5-year recurrence^4^					*0.090*
Recurrence free	58 (79)	40 (69)	14 (24)	4 (7)	
Recurrence	15 (21)	6 (40)	6 (40)	3 (20)	

^1^Percentage of the column.

^2^Percentage of the row.

^3^ *P* value based on Pearson's *χ* ^2^ test; ^∗^ *P* ≤ 0.05.

^4^Presence or absence of local or distant metachronous recurrence within 5-year followup.

^∗^Significant correlation between expression of cathepsin D at MTB with distant metastasis.

**Table 3:** Cox regression analysis of tumor characteristics with respect to cancer survival.

Variable	HR	95% CI	*P* value
Histological type (mucinous versus nonmucinous)	1.230	0.328–4.611	0.759
Histological grade (high grade versus low grade)	0.551	0.209–1.448	0.229
Vascular invasion (positive versus negative)	0.751	0.325–1.735	0.503
Operation (emergency versus elective)	1.079	0.467–2.492	0.859
Perineural invasion (positive versus negative)	2.788	1.055–7.370	0.039
Stage (I, II, III, IV)	15.333	6.030–38.992	0.0001
Cathepsin D expression at MTB (strong, moderate, weak)	0.986	0.577–1.684	0.958
